# The application and implications of science and technology innovation in the management of education for Chinese students studying abroad in China in the epidemic era

**DOI:** 10.1371/journal.pone.0306785

**Published:** 2024-08-16

**Authors:** Tiejun Zhu, Mengzhen Gu

**Affiliations:** 1 Institute of International Education, Anhui Polytechnic University, Wuhu, Anhui, China; 2 School of Design, Anhui Polytechnic University, Wuhu, Anhui, China; Islamic University of Madinah, SAUDI ARABIA

## Abstract

**Background:**

Although COVID-19 has brought serious disasters to all mankind, it has also accelerated the innovation and application of educational science and technology. China is the first to bear in COVID-19, and in order to minimize the impact of the field of education and teaching involving a large number of students, educational technology has entered a stage of continuous innovation and large-scale application, especially in higher education institutions.

**Purpose:**

Firstly, it introduces the progress and achievements of China’s educational science and technology innovation during the COVID-19 epidemic, as well as the in-depth application of it in education for studying abroad in China. Secondly, the problems and gaps of STI in education for studying abroad in China are analyzed. Once again, it puts forward the solution countermeasures and future development strategies for the science and technology innovation of education abroad in China.

**Methods:**

A cross-sectional survey of 258 international students from 20 universities was conducted using a self-administered questionnaire entitled "Research on Innovation and Application of Science and Technology in China’s International Education under the Situation of Conventional COVID-19 Epidemic Prevention and Control".

**Results:**

It will put forward opinions and suggestions to promote the deepening and improvement of China’s education science and technology innovation and application, as well as to provide a commentary and theoretical contribution to similar issues and phenomena around the world.

**Conclusion:**

Through in-depth research and analysis, it is found that the application of science, technology and innovation has played a great role in China’s study abroad education, which not only improves the quality and effect of teaching, but also enriches the means and methods of teaching.

## I. Introduction

As of June 2021, the total number of international students in China has exceeded 570,000, and their number has continued to grow for many years [1]. China has become an important destination for international students and the largest one in Asia. While the number of international students has reached a new high, China attaches great importance to the quality of their education in China. Not only the proportion of graduate students continues to grow, but also the discipline distribution is more reasonable. The number of students majoring in Chinese language has dropped from the previous second to the fifth place in 2021 [2].

COVID-19, which appeared at the end of 2019, is still in a large-scale epidemic situation all over the world. As the first country to encounter severe impact, China has accumulated and established a set of effective epidemic prevention and control system and corresponding social operation security system, of which an important link is the large-scale and effective implementation of educational scientific and technological innovation and "Cloud Education" [3]. Dai Wenqi [4] pointed out that due to the epidemic, the remote virtual classroom replaced the traditional offline teaching, and new types of classes such as live classes and recorded classes came into being. software such as Zoom and Classin became the teaching platform, and social software such as WeChat, Whats App, You Tube, Jitterbug, and so on, assisted in teaching. This achievement not only benefits from the "Internet + Education" strategy of the early layout of China’s government, to a large extent, it is also due to the comprehensive factors that caused the huge changes in the global political economy brought by the long time and massive COVID-19 epidemic. China’s remarkable achievements in COVID-19 prevention and control and the measures of providing substantive assistance to other countries have made China’s economy recover and rebound strongly as early as possible. It has also won wide praise in politics and diplomacy, and attracted many international students to study in China. Wang Siyang pointed out that “One Belt, One Road” has achieved great results since it was proposed in 2015. “The Outline of the 14th Five-Year Plan clearly states that we should continue to promote the high-quality development of the ‘Belt and Road’ and strengthen cooperation in science, technology and education [5]. It can be said that the attractiveness of studying in China has further improved with the country’s economic, scientific and technological development and comprehensive strength.

Based on the above background, taking international students studying in China as the research objects, and the development before and after the outbreak of the COVID-19 epidemic as the time course, this paper shows the innovation and application of science and technology of international education in China under the regular COVID-19 prevention and control situation, mainly reflected in: Before the outbreak of the COVID-19, China’s international education was still focused on quantity and scale, the classroom education and teaching methods were more traditional, and the practical teaching could be carried out in a down-to-earth manner; Liu Jiajie(2020) [6] pointed out that at present China’s internationalized education teachers are weak. The development of local internationalized education in China needs to be guaranteed by highly qualified teachers with rich experience in international education. In the early stage of the outbreak of the epidemic, there were many difficulties in the implementation of education and teaching, but can respond positively; After the epidemic trend stabilized, the innovation and application of science and technology of China’s international education quickly followed up, and the teaching contents and teaching methods were innovated. Although the practical open teaching was significantly reduced compared with that before the epidemic, the innovative practical teaching by using scientific and technological means was comprehensively promoted. Du Yinmei et al. (2023) [7] point out that through a number of thoughts on online education, it is believed that online education is more suitable as an effective complement to offline education as a mainstream supplementary learning tool.

Through the comparison between before and after the epidemic situation and between China and foreign countries, this paper also focuses on the challenges and limitations in the innovation of science and technology of international education in China, as well as the urgent improvement of educational science and technology. For example, although research and development achievements show explosive growth, there are not many high-end products, and the similarity of some product attributes is high, and there is the phenomenon of repeated research and development; Compared with developed countries, research and development funds are still insufficient; China’s colleges and universities are widely distributed, with a large number of international students enrolled in China, and the quality of talent training and the application of educational science and technology products are uneven by region and discipline; Some educational science and technology products have not been effectively developed for the learning and living environment and characteristics of international students in China, resulting in insufficient application validity and so on.

## II. Literature review

With the continuous growth of the scale of international students in China and the remarkable achievements China has made in the prevention and control of COVID-19 epidemic situation and the development of educational science and technology, many scholars have focused on this and carried out a series of studies. The main research views on the innovation and development of educational science and technology of China’s international education are summarized as follows:

A large number of articles focus on the implementation and application of various educational scientific and technological innovation achievements. Liu saie et al. (2020) [8] studied and discussed the design requirements, system user, system structure and implementation of the new online examination system. Wu Hao(2020) [9] focused on the function and security performance of continuing examination after the interruption of distance education online examination system. Wu Daguang et al.(2020) [10] pointed out that this large-scale online teaching in the preparation of teachers and students for online teaching, the support of the teaching platform, the technical services of the teaching platform, the online services provided by the school, the mode and characteristics of online teaching, the effect of online teaching, and the most important factors affecting the effect of online teaching. Huang Zuopeng et al. (2021) [11] discussed the strong technical support of 5G technology application for the pursuit of personalization and intelligence in Chinese education such as China’s international education. Wang Jinrong (2021) [12] carried out a feasibility study on AI technology in experimental teaching behavior analysis, and fully discussed the profound connotation of "intelligent" education in experimental teaching. Zhao and Li (2021) [13] studied the relationship between education, science, technology and social coordinated development from the perspective of complex systems. Yuan Qingjin (2021) [14] explore the research on the transfer of traditional teaching methods to e-learning in the Covid-19 pandemic from the perspective of Chinese students, and it discusses how online learning is being carried out in many countries and different types of online learning models are being promoted and implemented.Liu Guiyu (2022) [15] pointed out that we should explore the interoperability and integration of online and offline education with the application of new media and information technology: focusing on practical experience, guiding international students to actively participate in global governance with the concept of "empathy", and practically improving the ways and means of education and management of international students in colleges and universities.

New teaching contents and methods for international education in China based on COVID-19’s prevention and control and technological innovation. Liu and Zhang (2020) [16] discussed applying a risk assessment of the overall smart campus framework in terms of risk identification, assessment, disposal, and control to form a set of network security risk assessment methods that can be widely applied to the current overall smart campus frameworks. Wang et al. (2020) [17] constructed the scene of distance education and management based on 5G technology and HD video under epidemic prevention and control, and provided the smart education case of iFLYTEK. Ding and Li (2020) [18] proposed a Delphi method and AHP method combined with Borda ordinal value method to study the risks after returning to school under the COVID-19 epidemic. Shi et al. (2022) [19] designed a risk assessment methodology from a disaster management perspective using a stress-state-response model. The risk indices of disaster and system vulnerability were modeled through simple and appropriate weights, and they were combined into the overall risk of university resumption, and specific countermeasures to reduce the risk level were proposed. This not only protects public health and safety, but also has some practical significance in improving the evaluation and rational decision-making capacity of all parties. Huang Peng et al. (2020) [20] pointed out that after COVID-19, China’s online education enterprises showed a blowout growth. Every place actively explored the new mode of online education in the areas of K12 education, China’s international education, lifelong education and other sub areas. Zhu Guiping et al. (2020) [21] proposed an innovative online teaching program for basic engineering courses, including instructional design and lecture interaction methods, which is no less effective than traditional classroom teaching, and students utilize the online network to interact better. In response to the "Internet+" international students’ innovation and entrepreneurship education field, Wang Xinrui (2021) [22] proposed to achieve real-time updating of international student information and social needs information by means of science and technology. Xiao et al. (2021) [23] have built a new engineering AI + talent training community with integration of industry and education, inter-school interaction and multiple coordination. Wang Shumin et al. (2021) [24] pointed out that in the "post-epidemic era" when the Covid-19 pandemic in China has been effectively controlled and the Covid-19 pandemic outside China is still serious, the effect of online teaching is directly related to the promotion of the educational community of "Belt and Road". Zhou Ningning et al. (2022) [25] explored the combination of online live platform teaching+micro-teaching recording+social platform, according to the characteristics of the Microcomputer Principle and Interface Technology course for international students, and made full use of the online teaching platform supported by modern network technology to design a multi-dimensional online teaching session before, during and after the class.

Scientific and technological products refer to the products through research and development, used to meet people’s certain needs. In the field of education, technology products include but are not limited to multimedia teaching equipment, online education platforms, intelligent learning tools, etc. These products provide more possibilities for education through technological means. Comparative study on educational science and technology innovation between China and foreign countries. Liu Xiaoyan (2019) [26] discussed the sustainable development and impact of the education of Central Asian students in China from the aspects of China’s economy, education, science and technology, and points out the gap in education quality and scientific research level between international education in China and developed countries such as the United States, Britain and Japan. The application of scientific and technological products in education

Wang Zhiqiang (2019) [27] identifies characteristics of Israel’s innovation and entrepreneurship education ecosystem and its successful experiences. Yuan Fang (2020) [28] pointed out the application of science and technology products in education is mainly manifested in the aspects of: diversification of teaching means(online education platform); Personalized teaching(Intelligent learning tools, data analysis); Optimization of teaching management(Optimize the teaching management process and improve management efficiency and quality through scientific and technological means) have been applied in teaching, but still the emerging technology disciplines and international courses for international students need to be strengthened. Guo Feng’e (2020) [29] systematically analyzes and summarizes the science and technology innovation driving models of some developed countries and cities by using the case retrospective method and inductive deduction method, and finds that while there are commonalities between domestic and foreign science and technology innovation driving development models, there are also differences in the four aspects of the driving factors, industrial agglomeration, government policies, and the innovation environment; Xiong(2021) [30] studies the organizational forms of scientific and technological innovation and transformation in American universities and points out the differences. Rhonda Christensen et al. (2021) [31] introduced a replication framework for classifying studies conducted in the area of educational technology as a possible guide to conducting and reporting replication studies in the field. Rahmati Jafar et al. (2021) [32] presented teaching English with the help of technology has an effective effect size and has shown the success of this technology in language learning through the calculation and evaluation of statistical data.

Based on the concept of integrating mobile Internet technology with curriculum construction and the interactive intelligent teaching mode of Chinese Culture Overview course for international students, Liu Xiaona (2020) [33] broke the time and space restriction of cultural learning by using technology equipment which improves the effectiveness of cultural teaching. Wang Zhijun (2019) [34] pointed out the development from the learning theory in the digital age to the ontology of "Internet + education", stressing the scientific and technological role of the Internet to effectively connect the entire education system. Liu Bangqi (2019) [35] introduced the background of the emergence and rise of smart classroom, and combs the course from smart classroom 1.0 to smart Classroom 2.0 and then to smart Classroom 3.0 in detail. He discussed the platform architecture adopted in these three development stages to illustrate the supporting role of technology platform for the operation and upgrading of smart classroom at each stage.

Zhou Lei (2023) [36] mentioned that online video teaching, online teacher-student interaction, and virtual simulation experiments have explored the application of the intelligent teaching mode of "integration of reality platform, virtual platform, and interactive platform" in the physics experiment class of international students, which has solved the problem that undergraduate students cannot normally complete offline experimental courses. Analysis on the advantages and disadvantages, social impact of educational scientific and technological innovation, and research and judgment on the scientific and technological development of education for studying in China. Gu Xinxin (2021) [37] proposed to deeply cultivate various scenarios of education services, empower participants in education ecology, improve learning experience, provide better teaching, research, and services through innovative breakthroughs in products and technologies, always pay attention to people’s growth, and advocate the combination of big data drive and intelligent connection mode to give birth to a new "Smart Education" mode. With rich carrying modes and display forms, it begins to break the boundary between virtual and real, expands effective supply, and makes it possible to integrate learning resources, meet the needs of students and improve teaching quality. Guo Kailin et al. (2020) [38] proposed that the innovative development of international education in China is a booster to promote the construction of a community of shared future for mankind. It is necessary to build a high-level discipline group for China’s international education, carry out the online and offline hybrid teaching mode regularly, and support the docking of international online teaching platforms and curriculum resources. Yang Xiong (2021) [39] pointed out the serious phenomenon and problem of "Educational Involution" in the AI era in China, analyzed its root causes, and put forward solutions. Hao Dan et al. (2021) [40] analyzed the application and impact of AI in higher education, including China’s international education, from the perspective of learning effect and educational equity. Gazi Mahabubul Alam(2021) [41] suggests that higher education is generally not that active from the job market perspective, while online learning has in fact made education much more passive through the comparison of two groups of graduates. Nanik Hindaryatiningsih (2023) [42] argues that online college learning does more harm than good, and that several factors both impede and support the success of online learning, including the social factors of peers and parents, the integrity of the technological learning tools, and the physical and psychological readiness of the students and parents.

With the development of globalization and internationalization of higher education, the demand of students for higher education has dramatically expanded. In the education of overseas students, the application of scientific and technological products is mainly reflected in the following aspects, such as: Language learning tools(Use language learning tools to help international students improve their Chinese level),Online course platform(provide rich teaching resources to meet the needs of students’ independent learning),Virtual laboratory(achieve remote experimental teaching and improve students’ practical ability and scientific research level). In addition, a large proportion of students intend to study abroad, which lead to the flourishing of cross-border higher education on an unprecedented scale. Thus, many scholars have examined the factors that influence students to study overseas (e.g. Kim, Bankart, Jiang, & Brazil, 2018 [43]).

Hossler and Gallagher (2020) [44] stated that the three-stage model is appropriate for illustrating the process of deciding to study abroad. The three stages refer to choosing to study abroad or stay in their home country, and choosing a destination country for studying abroad and choosing an institution for higher education. Although this process is normal in decision making, some students purposefully choose the HEIs, directly bypassing the destination consideration (Chen, 2007) [45]. Most of the literature that analyses the process is affected by ‘push–pull’ theory (Lee, 1966) [46]. Generally, push factors are related to some negative aspects of the home countries that force the students to leave and study abroad. On the contrary, the pull factors are associated with the positive aspects of destinations that attract the students to study in other countries (Liu & Zhu, 2019) [47]. Push and pull factors attract students and motivate them to study abroad, which explain the determinants that cause outflow and are relevant to the first stage of the three-stage model, whether to study abroad.

To sum up, many scholars have shown the progress of China’s educational science and technology innovation under the regular COVID-19 epidemic situation and its in-depth application in international education in China. They have also carried out comparative research and revealed relevant problems, but there are still places for further deepening, mainly focusing on: first, there are a little literatures focusing solely on the science and technology innovation of international education in China; Second, there are more qualitative analysis and less quantitative analysis combined with special survey; Third, the macro research at the policy level is in the majority, and the panoramic display research combined with cases is lack; Fourth, the problem analysis is not comprehensive and in-depth, mostly pointing to a certain type of problems, and the evaluation, thinking and prospect of its social relevance and influence are insufficient.

Therefore, on the one hand, using qualitative analysis, this paper carries out empirical analysis in the form of questionnaires and interviews to show the actual situation of science and technology innovation of international education in China under the regular COVID-19 epidemic situation as far as possible. On the other hand, on the basis of quantitative research and case analysis, this paper demonstrates the above "research gap" and focuses on presenting a series of existing problems, social relevance and puts forward the countermeasures and future development strategy.

For the series of problems analyzed and sorted out, this paper will also deeply discuss and summarize them, and systematically put forward solutions and implementation plans on the basis of fully considering the advantages and disadvantages, so as to offer comments and suggestions for promoting the continuous deepening and improvement of the innovation and application of China’s educational science and technology, and provide evaluation views and theoretical contributions to similar global problems and phenomena. This paper takes the innovative application of science and technology in education for study abroad in China in the context of the normalization of the COVID-19 epidemic prevention and control as the research theme, aiming to explore the following issues:

(1)What impact did online education have on international students during the COVID-19 epidemic?

(2)How to assess the role of science and technology applications and innovations in international education in China in improving the quality of education and promoting equity in education?

(3)How to combine the application of science and technology and innovation in China’s international education with global education trends to promote the common development of global education?

In order to answer these questions, this paper systematically discusses the following four aspects:

(1)The progress and achievements of China’s education science and technology innovations during the COVID-19 epidemic were introduced, as well as the in-depth applications in education for students studying in China, such as the air classroom, virtual simulation teaching, and online teaching.

(2)Problems and gaps in science and technology innovation in education for studying abroad were analyzed, such as insufficient innovative power, limited financial investment, imbalance of regions and disciplinary specialties, uneven quality, and tendency of involution.

(3)It puts forward the countermeasures and future development strategies of science and technology innovation in education for studying abroad, such as re-examining the development pulse of the times, strategically laying out the application of science and technology innovation, re-evaluating the educational needs and scientific and technological resources, constructing a new ecology, re-positioning the safeguard mechanism of scientific and technological innovation, enhancing the protection of property rights, and establishing a dynamic evaluation and supervision system.

(4)To promote the continuous deepening and improvement of China’s education technology innovation and application, to make comments and suggestions, and to provide analytical perspectives and theoretical contributions to similar issues and phenomena around the world.

## III. Research methodology

### 3.1 Research object

The researchers conducted a questionnaire survey on the innovation and application of international education science and technology in Chinese colleges and universities. Before the research was conducted, the researchers issued a recruitment notice to recruit international students to participate in the questionnaire. These participants came from 20 colleges and universities, with a total of 258 people. Among them, there are 163 female students and 95 male students. We sent written informed consent forms to each participant to obtain their consent. This study did not include minors. The author collected data on January 21, 2022, and accessed the data for research purposes on February 28, 2022. Author had access to information that could identify individual participants during or after data collection.

### 3.2 research methods

**Two types of research methods are conducted in total, there are:**

First type: Questionnaire (2.1) with five parts, containing participants personal information, the online platform used in the class, the main problems in the face with educational technology products and intelligent teaching, acceptance of intelligent educational science and technology and related products, and ect., show the common challenge and problem that students face in the innovation and application of technology in international teaching.

Second type: Case Studies (2.2) contains Air classroom, Virtual simulation teaching, Frontier achievements in mental health education and psychological science research, Popularization and application of COVID-19 epidemic prevention and control, and educational science and technology products.

### 3.3 Research tools

A self-developed questionnaire on "Research on Innovation and Application of Science and Technology in China’s International Education under the Situation of Conventional COVID-19 Epidemic Prevention and Control" was used, with a 6-point Likert scale, where 0 stands for not in favor of it at all, and 5 stands for in favor of it at all.

The questionnaire consisted of five parts. The first part is the relevant information of the respondents: age, gender, current university, year of study, describe their IT skills, and specify if they had previously participated in any online courses and so on.

In the second part, international students were asked to determine the format of the materials offered by their schools, identify ICT devices, use online platforms as a means of e-learning, and also the amount of learning time and learning efficiency during online classes.

The third part aims at the main problems when international students face to the educational science and technology products and intelligent teaching. Then the international students need to determine their level of learning before and after the epidemic and through e-learning.

In the fourth part, international students were asked to rate the level of acceptance of intelligent educational science and technology and relevant products using the Likert scale from 1 to 5 (1: extremely unenjoyable and 5: extremely enjoyable). Students were also asked to rate smart classroom activities (1: extremely inactive and 5: extremely active).

The last part mainly included the positive and negative perceptions and corresponding suggestions.

School of International Education of Anhui Polytechnic University provides ethical approval for the research in the manuscript and all respondents agree to use questionnaires to collect data from them in this study.

## IV. Results

### 4.1 Questionnaire

According to the first part of questionnaire, the basic information of the respondents showed in Table 1. It shows that 58% of respondents were male and 42% were female. The majority of the respondents (68.4%) were undergraduate students, and (31.6%) were postgraduate students. The plurality of the respondents (73,7%) were studying in public schools, whereas (26.3%) were in private schools. All the respondents (100%) were between 18–25 years old.

**Table 1:** Basic information of the respondents.

ITEM			PERCENTAGE	
Gender	Male	Female	58%	42%
Education level	Undergraduate	Postgraduate	68.4%	31.6%
Nature of School	Public	Private	73.7%	26.3%
Age	18–25 years old		100%	

#### 4.1.1 Data analysis

**Fig 1:**
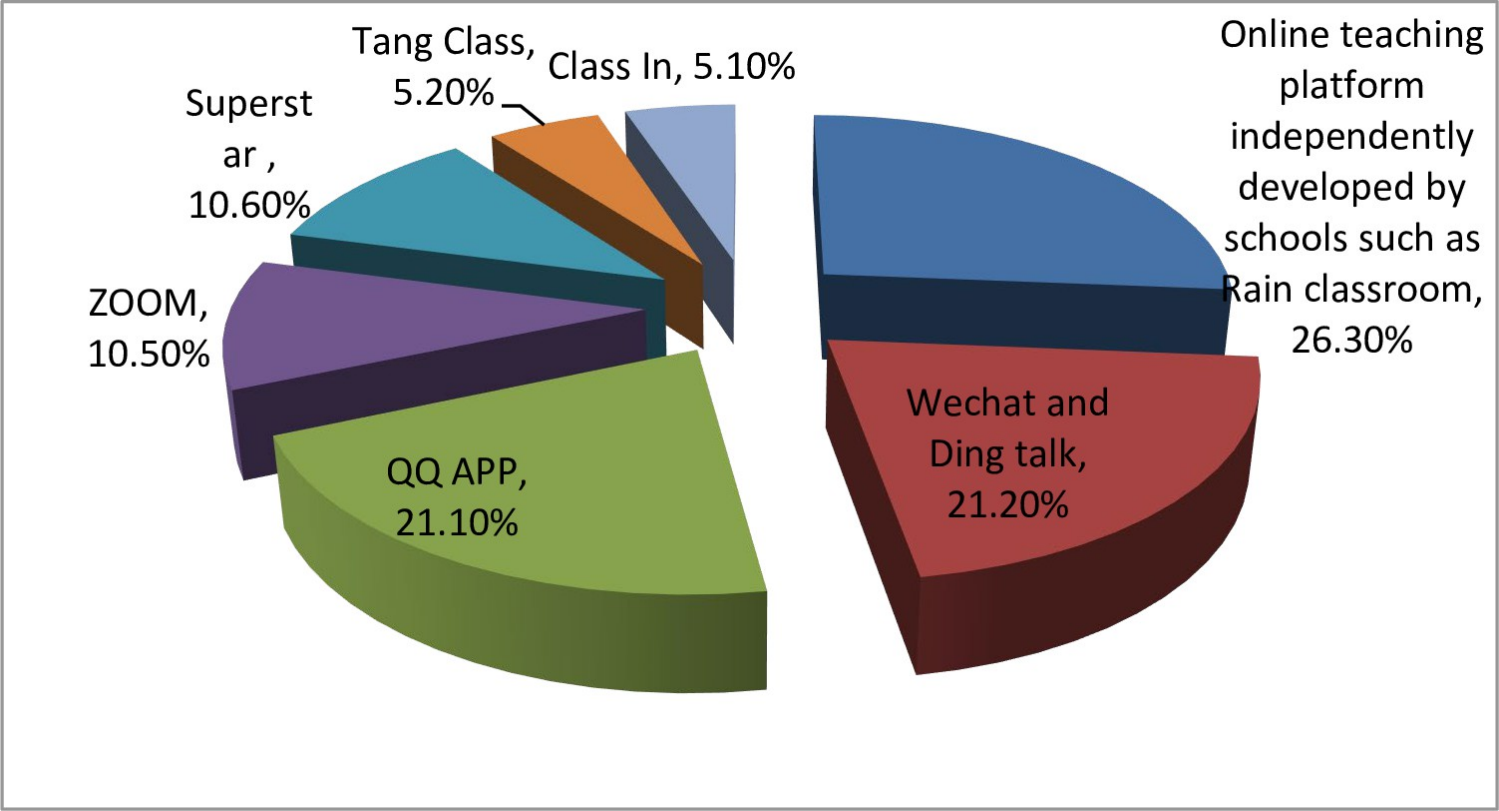
Online teaching platforms used for online classes.

*ICT device and online teaching platform data analysis*. As can be seen from Table 2, 84.2% of international students take classes online. Prior knowledge of information technology (ICT) is required for online courses, and the data revealed that 85% of the students had prior knowledge of ICT. The majority of the students (47.3%) use laptop and computer for taking online classes, only 21.1% of respondents use mobile phones completely. The reason is that accessing online courses through mobile phones will divert student learning attention to a certain extent, because it is difficult for them to control not to open social media sites, information push of relevant APP and checking messages, so they will lose some interest in the ongoing course. A total of 47.4% of the students said connectivity of their internet is poor which led to disconnection during the online classes more than 3 times for 57.9%, 2 times for 5.3% and for one time 36.8%. Moreover, 89.5% of the students faced time difference problem. Fig 1. shows that the online teaching platform independently developed by the school accounts for 26.3%, whereas 21.1% of schools used QQ APP. The third online teaching platform is ZOOM, accounting for 10.5%. The rest of school are using Wechat, Ding talk 21.2%, Superstar 10.6%, Tang Class 5.3% and Class In 5.3%.

**Table 2:** ICT device knowledge of the respondents.

ITEM	ANSWER			PERCENTAGE		
Taking online classes	Yes		No	84.2%		15.8%
Prior knowledge of ICT device	Yes		No	86%		14%
ICT devices used by Students	Laptop Smartphone Both laptop and Smartphone Computer			36.8% 21.1% 31.1% 10.5%		
Quality of Internet	Best Good Poor			15.8% 36.8% 47.4%		
Disconnected during online classes	1 time 2 times More than 3 times			36.8% 5.3% 57.9%		
Time difference problem	Yes	No		89.5%	10.5%	

*Student’s negative and positive perceptions*. Abruptly, the education system shifted toward massive online classes due to the COVID-19 epidemic. Hence, it is necessary to know about the opinion of international students towards online education. The researchers asked both the positive and negative perceptions of intelligent teaching mode from the international students in China.

***A*. *Student’s negative perceptions*.** Table 3 represents negative feelings, demerits and opinions of the students towards the application of educational science and technology which were asked by researchers on three-point Likert scale statements as satisfied, not satisfied and comparatively. The highest response rate was 94.7% as the students said that long-time online classes are not comfortable when compared to offline classes. The majority of the respondent students (63.2%) said that they are not learning as much as they were before the COVID-19 epidemic. A total of 83.3% of the students opined that there is a low motivation for study in online classes due to a lot of distractions at home, while 66.7% of the respondents did not understand the content delivered in online classes. In online classes, students felt isolated because there are no real group projects, lack of communication and restriction in outdoor activities, which leads to social isolation. 33.3% of the international students were not interested in online courses because of the problem of internet connection in class.

**Table 3:** Students’ negative perception.

NO.	STATEMENT	PERCENTAGE
1	Long-time online classes are not comfortable than offline classes	94.7%
2	Low level of learning	63.2%
3	Low motivation for study in online classes	83.3%
4	Not understand content delivered in online classes	66.7%
5	Disinterested to take Online Classes because of internet connection problem	33.3%

***B*. *Student’s positive perceptions*.** Table 4 shows positive opinions of the respondent students. Due to the flexibility of time and space and no transportation cost, air classroom has become a convenient choice. All students (100%) believe that online courses save transportation time and costs. The options with high student evaluation (33.3%) are the development of online teaching mode and the application of educational science and technology products, which effectively improve students’ scientific and technological literacy. 33.3% of the students chose the positive option of "getting more knowledge in online courses". In addition, a total of 78.9% thought it exciting to attend online classes with people from all over China and lots of other countries.

**Table 4:** Students’ positive perception.

NO.	STATEMENT	PERCENTAGE
1	Online courses save transportation time and costs	100%
2	Improvement of students’ scientific and technological literacy	33.3%
3	It’s exciting to have online classes with people from all over China and many other countries.	78.9%
4	Get more knowledge in online courses	33.3%

*Comprehensive average value of international students’ positive and negative perceptions*. Table 5 shows the mean values for all positive and negative perceptions of the respondent students towards the innovation and application of international education science and technology. The mean value for the positive feelings and perceptions of international students is 3.79, which is higher than the mean value with 2.56 of negative perceptions. Therefore, international students on average have a positive attitude towards the innovation and application of international education science and technology, as it has emerged as a bridge to reduce the learning gap due to school closures and help international students maintain their academic interest and development during the ongoing COVID-19 pandemic. The positive result is that a total of 57.9% of the respondent students like new technologies because they provides new learning modes and new methods to solve problems. Whereas 42.1% believe that face to face learning is the best solution.

**Table 5:** Comprehensive average value of international students’ positive and negative perceptions.

DESCRIPTIVE STATISTIC	MINIMUM	MAXIMUM	MEAN
Negative Perception	1	5	2.56
Positive Perception	1	4	3.79

#### 4.1.2 Analysis results

*Problems and analysis at the micro-level*. Technology is a double-edged sword. It plays an important role as a booster in the process of improving the quality and efficiency of international education, teaching, and management. For individual foreign students, they can better contribute to autonomous learning and personality development, but they may also aggravate educational differentiation and lead to educational injustice and imbalance. If individuals rely too much on the power of science and technology, they will weaken their learning ability and research spirit. In addition, there are still many areas where technological software and hardware, educational content, and teaching details need to be continuously improved. Therefore, while seeing the remarkable achievements in the innovation and application of international education science and technology, we should also pay attention to and think about its existing problems.

According to the feedback on the disadvantages of international education science and technology innovation and application for international education set in the questionnaire, the details and analysis are show as follows:

Table 6 shows major challenges confronted by the international students while pursuing online classes during the COVID-19 pandemic.47.3% of the respondents admitted that the lack of effective supervision would cause online learning to be inattentive and inefficient. A total of 31.2% of respondents thought they had difficulties in demonstrating their learning in online classes and 17.4% respondents faced difficulties in submitting their assignments in online classes due to connectivity issues and device problems. Moreover, 20.1% of the international students had difficulties in attending online exams.

**Table 6:** Challenges faced by international students in online classes.

NO.	CHALLENGES	PERCENTAGE
1	Lack of effective supervision would cause online learning to be inattentive and inefficient	47.3%
2	Demonstrate their learning	31.2%
3	Submitting assignments	17.4%
4	Attending online exams	20.1%

The researchers set up a free item to let respondents talk about their experience with online classes during the COVID-19 pandemic. After careful reading and analysis, here are the general challenges and problems faced by the students with the innovation and application of international education science and technology, which shows the biggest six problems about foreign students studying in China through on-line courses.

***A*. *Adaptability*.** Due to the rapid spread of COVID-19 and the large-scale and rapid application of online teaching and educational technology products, for international students who need to adapt to many links such as language, culture and educational methods, this change is really difficult to adapt quickly.

***B*. *Technical issue*.** Many international students are not well equipped with the high internet connection required for online learning, especial the students from less developed countries and regions. As a result, they face problems with virtual learning live broadcasts and other platforms that require an internet connection. They have technical problems because they are not very familiar with technology and computer applications. A slow and high internet connection can play an important role in how quickly you can attend class and not miss any live sessions.

***C*. *Scientific and technological application knowledge and ability*.** The use of many new equipment, scientific and technological products and intelligent software and platforms requires high scientific and technological application knowledge and ability and corresponding training. And whenever some technical issues arise, they find it difficult to solve the problem in such a scenario. They face difficulties in live classroom, use of appropriate codes, artificial intelligence system operation software, communication related applications and websites, browsing of study materials and so on.

***D*. *Self-motivation*.** For international students who are prone to poor social communication, due to the relatively isolated learning environment and lack of interaction of online education, they will soon lose their confidence in learning as soon as they find it difficult to learn online. It requires motivation to complete tasks and to involve students in their learning. Lack of motivation is a common challenge for all international students.

***E*. *Distraction*.** Online learning at home is a complex experience. You might expect things around you to be like a school campus. But things are different at home, for example, you may want a huge classroom, gardens, playgrounds, canteens, friends and teachers around you to guide and learn. But online learning, you have to manage everything in one room with parents around you, or even only yourself. You can be easily distracted by the little things in the house.

***F*. *Teaching and learning styles*.** The application of novel online teaching platform and intelligent education technology products has changed the traditional form and part of the content of teaching and learning to a great extent, even the teaching and learning habits and styles. For international students, due to language, communication and other reasons, they can obtain relatively few online resources, so these changes will make it difficult for them to obtain learning resources and information equivalent to those obtained under the traditional learning mode in a short time.

***G*. *Virtual engagement*.** Online teaching platform help teachers provide learning materials and assignments, communicate via email, live chat or messaging, and deliver content via live sessions, presentations, recorded videos, or lectures to students. Despite all these activities, some students still do not find participation compared to the traditional activity. Students find it difficult to communicate in person who have difficulty understanding concepts. Often these students do not approach the teachers to clarify their doubts.

***H*. *Feedback*.** Every student needs feedback about their performance during the learning process so that they can improve their learning abilities. It is not only noticed during exams but also for every task and project. The feedback information from the questionnaire shows that international students rarely seriously look at their homework correction, or the suggestions, comments and messages of teachers or students on the online teaching platform. Therefore, the online feedback mode is difficult to obtain the same effect as the offline evaluation.

*Problems and thoughts at the macro-level*. The questions statistically analyzed in the above questionnaire reflect the relevant demands, experience, perception and satisfaction of individual international students in China, representing problems and deficiencies at the micro-level. But in fact, what is involved and related behind this is the relevant deficiencies and weak links at the macro-level, mainly reflected in:

Scientific and technological innovation force of international education in China is insufficient, the talent team is insufficient and the structure is unreasonable; The investment of educational science and technology innovation funds is limited, which does not meet the needs of education, and the distribution and use is not very reasonable; The innovation and application of international education science and technology in China are unbalanced in regions, disciplines and majors, with great differences and insufficient synergy; Although there are research results that are in line with the international frontiers and advanced, the number is limited and the application is not yet mature. The continuous guarantee policy is not enough and needs to be further improved; The quality of international educational scientific and technological innovation achievements is uneven; The characteristic application of colleges and universities combined with practice is not enough; The application feedback of international students and related aspects is not enough; The rapid development of science and technology, big data of educational information, resource sharing and network communication have led to serious involution in China’s basic education, and there is also a tendency and sign of involution in the China’s international education; The role of educational science and technology may be alienated. If it is not used properly, it will become a cold tool to measure people’s ability and efficiency, and help management and educators more rigorously supervise and control educational objects.

China’s education is criticized for exam-oriented education. At present, all parties are trying to make changes to effectively change this situation, and educational scientific and technological innovation is the most striking scientific path. However, just as Yang Dongping, professor of the Institute of education of Beijing Institute of technology, sharply commented and questioned: "we have a lot of educational innovation in real life today, is it subverting and changing exam-oriented education, or is it providing more refined exam-oriented education and comprehensively binding teachers and students with big data? " Under the policy guidance of assimilative education and management, whether the education of international students in China will fall into the quagmire of "scientific and technological exam-oriented education", and how the Chinese government, schools at all levels and international students without exam-oriented education background should face and solve these are worthy of vigilance and reflection.

### 4.2 Case studies

This part introduces the achievements of China’s educational science and technology innovation during the COVID-19 epidemic, as well as the in-depth application of it in education for studying abroad in China.

#### 4.2.1 Air classroom

Under the situation of COVID-19 epidemic prevention and control, online teaching has become the norm in China’s international education. According to incomplete statistics, there were more than 700 colleges and universities recruiting international students in China in 2021. According to the policy of "Suspended class, ongoing learning" issued by the Ministry of Education in February 2020, colleges and universities recruiting international students in China overcame various difficulties and opened ""Air classroom" for all international students. For example, the "Air classroom" for international students of Anhui Medical University opened for the first time on February 24, 2020. There are 205 international students on campus, and 213 people are abroad. International students are distributed in more than 20 countries such as South Africa, India, Jamaica, Malaysia, Tanzania and Papua New Guinea and so on. International students on campus watch the live courses on time. Considering the time difference, international students abroad can watch live broadcast or video courses stored on the online platform. There are many intelligent teaching platforms used in the air classroom, including "Rain classroom", "Dingtalk live broadcast", "Superstar" and "Tencent classroom". Some colleges and universities with strong scientific research strength have also developed and issued separate online teaching platforms. In view of the fact that some domestic online teaching platforms do not have English versions or cannot be applied abroad, some colleges and universities have also adopted foreign online teaching platforms, such as ZOOM, WEBEX, and Teams. In addition, the online education implemented in China is not only online classroom, but also realizes online implementation of all links such as online course introduction, online discussion, online homework, online correction, online test, online defense, online evaluation, online analysis of course effect, online academic lectures, online training, online conference, online collaborative development of courses, online data resource sharing and so on. For instance, in March 5, 2020, Dalian University of Technology applied the "TANG" international education cloud platform to organize the entrance examination for language freshmen from five continents [48]. Hebei University and Tianjin University have used the "Internet+" international education cloud platform to carry out online collaborative development and online teaching resource sharing of the intercollegiate course of "China Overview". The educational technology innovation consciousness and strength behind this are unprecedented, and the difficulties that are overcome behind it are not only scientific and technological research, hardware and equipment update, a large amount of funds, but also involves the allocation and management of human resources such as technical training, teacher learning and online application. After the epidemic has been effectively controlled in China, the mode of online teaching has been deeply rooted in the hearts of the people. The government and schools have generally launched a hybrid teaching mode combining online and offline according to the advantages and disadvantages summarized after the large-scale implementation of online teaching. It can be said that online teaching has become an indispensable highlight and feature in China’s international education.

#### 4.2.2 Virtual simulation teaching

In the context of regular COVID-19 prevention and control situation, the biggest difficulty in the process of online teaching and international students’ education and cultivation lies in open practical teaching. In the early stage of the epidemic, the practice course was suspended in a large area due to the severe situation of the epidemic, but this measure can only be used as a short-term response, because if the practice teaching is missing, the quality of talent cultivation will be greatly reduced. Therefore, the Chinese government, universities and scientific research institutions attach great importance to this urgent bottleneck. On the one hand, they speed up the implementation of the national virtual simulation experiment teaching project to be built in 2018. On the other hand, they take this as a breakthrough point to carry out scientific research, optimization, improvement, promotion and application in combination with the epidemic situation and current reality. Virtual simulation teaching mainly relies on technologies such as sensing technology, man-machine interface technology, computer graphics, etc. in fact, they all belong to the well-known virtual reality, augmented reality and artificial intelligence, which have been used in some schools and educational institutions. The three-dimensional virtual reality world constructed by the virtual reality system composed of professional graphics processing computer, virtual reality application software and input and output equipment can provide students with real experimental scenes and experience effects. With augmented reality skillfully and flexibly superimposes fictional information and real environment through computer simulation technologies and simulation effects such as image matching, pose tracking, pattern recognition, realistic rendering and machine learning, so as to realize the interactive integration of virtual and real, and make students feel like they are on the scene. It is an optimal alternative for students to carry out experimental operation, skill training, even graduation practice under the influence of the epidemic. Taking Civil Aviation University of China as an example, the university has one national virtual simulation experiment center and two Tianjin municipal virtual simulation experiment centers, which are open to Chinese students and international students. Among them, the national virtual simulation experiment teaching center of aircraft mechanic maintenance engineering has built a series of experimental teaching systems represented by multi-functional aircraft mechanic maintenance training system, open cockpit engineering platform, digital twin fleet and a highly simulated one-stop 3D virtual experiment environment. Students can realize the strong reproduction of the real scene here without going to airports and other places with high epidemic risk. In the laboratory, a series of sensing auxiliary facilities such as stereo glasses and sensing gloves can be used to realize the visual, auditory and tactile perception of the real scene, and carry out multi-level virtual simulation practice teaching of aircraft mechanic maintenance, such as immersive and communicative industry cognition, maintenance foundation, system synthesis, maintenance engineering and innovative training; Based on the virtual simulation experiment teaching platform, Hubei University of Science and Technology carries out the course teaching of Systematic Anatomy with heavy practical tasks and high requirements for international students majoring in medicine. Through the learning and virtual simulation experiment of five-unit modules of motor system, visceral science, vascular system, sensory organ and nervous system, international students can "operate on the spot" without leaving the school. Moreover, the university also conducted a comparative experiment, according to the test results of the experimental group and the control group, the international students who use virtual simulation experiment teaching have better academic performance and learning efficiency; Peking University, Tsinghua University and Zhejiang University, in cooperation with AI vision company SenseAR, are carrying out "AI + AR" software and hardware and industrial ecological development layout, and are applied in higher education, including China’s international education. For example, by integrating augmented reality interaction with course content, SenseAR special effect engine helps educational institutions build smart virtual classroom through mature AR and AI technologies including make-up mode, background segmentation, special effect stickers, gesture detection and so on. With the green screen technology, the classroom scene is no longer limited to the traditional blackboard or projector, can be transformed according to different course contents, add relevant AR virtual elements, and adaptively integrate teachers with rich teaching scenes.

#### 4.2.3 Introduction of frontier achievements in mental health education and psychological science research

In addition to bringing the impact of learning and life on the students, COVID-19 has also affected and disturbed their psychology, especially those international students who have been abroad for a long time. They not only have the fear of large-scale COVID-19 epidemic, anxiety about their learning and life, but also worry about the health and safety of their families. Therefore, the psychological counseling and mental health education for international students are even more important. In this regard, on the basis of ensuring the normalization of the study and life of international students, Chinese colleges and universities fully consider the psychological problems faced by them, such as the impact of the epidemic, language barriers, cultural conflict barriers and interpersonal communication barriers and so on. On the one hand, through setting up mental health education courses, carrying out student activities, providing personalized psychological consulting and instruction services, strengthening psychological intervention and psychological rehabilitation to solve all kinds of psychological problems of international students in time. On the other hand, China’s colleges and universities also pay attention to introducing the frontier achievements of COVID-19 prevention and psychological science, and carry out psychological experiment analysis and case studies in order to facilitate the accurate implementation of psychological diagnosis and enhance the therapeutic effect. For example, the pathology department of the First Affiliated Hospital of Anhui Medical University analyzed the pathological anatomic report of the first novel coronavirus pneumonia death case in the world published in the global top medical journal "Lancet" for all international students. Let them understand the novel coronavirus pneumonia knowledge, infection route, clinical manifestation and preventive measures, and then enhance their psychological cognition and eliminate nervousness. University of Chinese Academy of Sciences proposed for the first time in the world that soft neurological signs are the internal phenotype of schizophrenia and a series of studies have been carried out, which is conducive to the early identification of schizophrenia. In the teaching of international students, the clinical application results of high-precision functional connectivity group in human cerebral cortex were introduced. The university also studied and applied the mode and key technologies of post disaster psychological assistance such as COVID-19, and put forward the mode of China’s post disaster psychological rescue. The researchers also worked with AI technician to develop and improve the emotion detection bracelet and adaptive psychological service system, which can be used to detect anxiety and depression among international students and provide corresponding psychotherapy.

#### 4.2.4 Popularization and application of COVID-19 epidemic prevention and control, and educational science and technology products

Under the regular COVID-19 epidemic situation, science and technology enabled China’s international education has entered the era of large-scale online teaching and intelligent classroom. In addition to the comprehensive innovation in teaching concept, teaching management, teaching mode, teaching content and teaching form, intelligent and novel epidemic prevention and control products, education and teaching technology products have also become highlights and features (Table 7). In the field of epidemic prevention and control, due to the massive outbreak of COVID-19 in China, a large number of university scientific research forces have carried out urgent scientific research in response to their characteristics and actual conditions. The research and application of epidemic prevention and control technology products are also the earliest in the world, such as medical sewage waste treatment equipment and disinfection robot urgently researched and developed by Jiangsu University; Automatic intelligent traditional Chinese medicine decoction system independently developed by Wuxi Research Institute of Nanjing University of Aeronautics and Astronautics; New thermal imager for abnormal body temperature detection developed by Nanjing University of Science and Technology; 5G network telemedicine diagnosis intelligent traditional Chinese medicine system developed by Shanghai University of Traditional Chinese Medicine; Intelligent digital stethoscope developed by Guangdong University of technology and so on. In addition, the scale of Chinese college and university students is generally more than 10,000 [49]. Therefore, Chinese colleges and universities are key places where large-scale people live for a long time. Epidemic prevention and control should complete all links of education, teaching and campus management in a non-contact manner as far as possible, so as to effectively reduce the risk of infection. Therefore, Chinese colleges and universities generally adopt the integrated access control system of face recognition gate and infrared thermometer, and set up "smart campus online service hall", international students can use the university relevant application systems and data resources when they are abroad, and enrollment, graduation and employment procedures can also be handled online; Use the smart campus epidemic prevention and control and health management platform to scientifically and accurately manage the travel and health data of teachers and students to and from the campus. In terms of international education science and technology products, new high-tech teaching instruments and learning equipment emerge one after another, which has become a learning partner for international students. For instance, simultaneous interpreter, it can achieve simultaneous interpretation with high accuracy and low price, providing great convenience for international students’ study and life in China; For another example, the intelligent recording pen developed by IFLYTEC can not only scan words and translate, but also play Chinese spelling, which is of great help to international students learning to use Chinese; Another example is the AR digital textbook of Chinese culture course, which transforms the traditional textbook into multimedia video or animation with the help of AR technology, so that international students can no longer learn Chinese culture monotonously during the epidemic, and can feel the breadth and brilliance of Chinese culture without leaving home.

**Table 7:** List of main products of China’s epidemic prevention and control, international education science and technology.

NO.	EPIDEMIC PREVENTION AND CONTROL TECHNOLOGY PRODUCTS	MAIN FEATURES	CHINA’S INTERNATIONAL EDUCATION SCIENCE AND TECHNOLOGY PRODUCTS	MAIN FEATURES
1	Nanometer photo-catalytic material degradation disinfectant	Continuously absorb visible light energy and produce a catalytic reaction, thereby killing all kinds of bacteria and viruses continuously and long-term	simultaneous interpreter	Voice recognition, simultaneous translation, voice shorthand, voice intercom
2	Thermal imaging human body thermometer	Infrared thermal imaging human body temperature measurement	Intelligent recording pen	Scan word search, scan translation
3	Hospital contactless self-service machine	Using interactive holographic air imaging technology, all processes such as appointment, registration, and payment can be completed without touch	AR digital textbook	Use AR technology to transform traditional textbooks into multimedia videos or animations
4	"5G+AI" intelligent security robot	Autonomous patrols on campus to reduce the risk of cross-infection caused by personnel mobility	Smart classroom interactive blackboard	Smart eye protection, smart recognition, touch interaction, screen writing
5	Gesture touch water dispenser	Three-dimensional gesture recognition control	Smart micro-lecture tool	Smart microphone and smart micro-lecture server, professional micro-lecture teaching resources can be recorded without special multimedia classroom
6	All-terrain spraying and virus killing robot (UAV)	Complete virus killing and epidemic prevention and control within 8,000 square meters in 5 minutes	Smart display board	Digital display, smart demonstration
7	Mask face recognition gate	AI recognize the face of a person wearing a mask	"Old China Hand" smart learning machine	All-round wisdom shows China’s geography, humanities, history and culture, etc.

## V. Discussion

"COVID-19 has had a great impact on the global social production and life order and macroeconomic situation". With the remarkable results of China’s epidemic prevention and control and the strong rebound of the economic situation, as well as the large-scale application of China’s educational scientific and technological innovation and online education, many international students choose China as their destination country. According to the "Questions to ’two sessions’ from foreigners living in E China" made by SuperChinese from March to April 2020 [50], the questionnaire has received 1090 valid responses from 25 countries, of which 72% are from Asia, 17% from Europe and 5% from America. Students aged 18–21 are the main respondents of the survey. Through data analysis, 48% of respondents made it clear that their study abroad plan was affected to varying degrees due to the epidemic, and 74% of respondents intended to choose to study in China in 2020 or 2021. In addition, according to the survey data of China Prospective Industry Research Institute, "In 2000, a total of 1.6 million students worldwide received overseas education, while China did not even reach 1%. In 2020, the number of international students who chose to study in China reached 9%, ranking fourth."

According to the research of this paper and the statistical analysis of the questionnaire data, China has made rapid development in China’s international education, which is due to the first is the government strategy and rapid and comprehensive deployment and implementation, the second is the international education and teaching concept and school running goal of improving quality and efficiency, and the third is that the COVID-19 epidemic has brought both impact and opportunity, with good prevention and control effect, economic development is beneficial to the whole world. Fourth, educational science and technology has developed rapidly, especially with fast application, large scale and good effect; It has formed a Chinese model and method, and expanded the global influence of China’s international education.

### 5.1 The great challenge of the COVID-19 epidemic to the educational program of studying abroad in China

While making rapid progress, a series of problems will inevitably arise. The main causes are as follows: First, it is difficult to make a comprehensive breakthrough in the traditional fixed mode and concept of Chinese education for a long time, especially the influence of exam-oriented education. Second, compared with developed countries, economic support and development still needs to be further improved, not only the total per capita is small, but also the investment in education needs to be greatly increased, especially in the central and western regions; Third, the number of international students is relatively large, however, the scale of intellectual resources and data in the China’s international education industry is limited and the type is relatively single as well, the sharing and integration of data is insufficient, and the low reserve and some isolated data make it difficult to deepen the integration of high-tech such as artificial intelligence. Fourth, technological radicalism is inevitable, and phenomena such as utilitarian kidnapping and value imbalance may occur; Fifth, security problems caused by technical vulnerabilities, including privacy disclosure and so on.

On the one hand, the development of informatization of education and teaching requires corresponding changes in the teaching mode. In the face of the epidemic of new coronary pneumonia, the traditional classroom-based teaching mode can not be carried out smoothly. In response to this situation, "online teaching carried by network platform" has become the main way of innovative practice, which integrates "Internet+", intelligent technology and other technical elements, and has successfully become an important development direction for Chinese and international higher education. It has successfully become an important development direction for Chinese and international higher education. However, with the rapid development of education informatization, some institutions of higher education are not fully prepared for the corresponding education concept, platform layout, personnel training and quality control when facing the informatization teaching program. In terms of educational philosophy, teachers in some colleges and universities are still stuck in traditional classroom teaching, failing to grasp the development situation in the face of big data and the Internet era in depth, and their teaching ideas are still stuck in the traditional indoctrination learning mode, and they have not yet practiced new teaching methods such as blended teaching and online education, and they lack the positive actions and energy to cope with the new changes of informationization in education and teaching. As for the problem of platform construction, some colleges and universities still rely too much on third-party communication platforms for online teaching, and have not yet established teaching platforms with complete functions and safety performance. The teaching process is often stalled due to platform congestion, network lag or online overload, resulting in unsatisfactory teaching results. In terms of teacher team building, some college teachers still fail to fully grasp the skills and functional characteristics of online teaching platforms, which makes it difficult for them to adapt to the online teaching environment, and it is difficult for them to reflect and give full play to the innovativeness of their teaching programs. In the process of monitoring teaching quality, some colleges and universities have failed to establish an appropriate online teaching quality assessment and monitoring system, which makes it impossible to accurately understand the actual results and knowledge level of students in participating in learning activities, and thus makes it difficult to effectively promote the continuous improvement of online teaching quality.

On the other hand, the requirement of service management refinement has gone beyond the adaptability of the traditional management mode. Under the influence of the epidemic, international students are unable to return to their home countries to receive normal education, which has led to a great challenge to the on-campus education and management mode, and the education department needs to rely on the Internet to provide international students with sufficient education support by means of the new education management system represented by the new class management mode and student organization management mode. With the increasing trend of internationalization, the scope of countries and regions involved in China’s international students has also expanded, and there is a practical challenge to improve the management service level of colleges and universities. Objectively, due to the obvious differences in the level of development of each country and region, the current status of network infrastructure construction is very different, and even some regions have not yet built smooth network coverage, so it is difficult for education managers to comprehensively cover all the students through an online meeting or online teaching, especially in different countries and time zones, and it is even more difficult to achieve timely and comprehensive! It is difficult to upload records and answer questions. Subjectively, in the post-pandemic era, educational administrators in many universities have not yet fully adapted to the trend of using the Internet as the main means to provide educational services for international students in China. There are also some deficiencies in the organizational mechanism and contact channels to cope with this trend, which have led to the failure to establish peer-to-peer network contact with all international students and provide them with timely and effective educational services, thus seriously affecting the quality of education services for students studying in China.

### 5.2 Response strategies for study abroad education in China in terms of improving the quality and equity of education by using science and technology application

In view of the weak links in the innovation and application of science and technology for China’s international education and the root problems of Chinese education involved, the Chinese government and all sectors of society are constantly trying to change and improve. According to the budget data of 2021 released by the Ministry of Education, Ministry of Science and Technology and other government departments, the budget for China’s international education in 2021 is 2, 982.217 million RMB [51]. The research and development budget of science and technology in 2021 is 53.8 billion RMB, an increase of 12.42% over the amount implemented in 2020, of which 14.23% is mainly used for the construction of scientific and technological innovation bases [52].

Firstly, at the policy level, the Ministry of Education of China has issued a series of policies, on the one hand, calls for continuing to strengthen educational science and technology innovation, "actively exploring the integration of online and offline education in the post epidemic era, and promoting ’Internet+’ conditions to reform and innovate the educational concept, mode, curriculum, teaching materials and teaching methods of China’s international education" [53]; Focus on online sharing of high-quality courses, the Ministry of Education collects and develops rich and high-quality online education and teaching resources, and provides students with high-quality special education resources free of charge by using the national and local education and teaching resource platforms and high-quality school network platforms, so as to promote the balanced development of educational resources and educational equity; Comprehensively strengthen the quality construction of China’s international education, and effectively solve the difficulties of insufficient and unbalanced innovation and application of educational science and technology in different regions and disciplines. On the other hand, the colleges and universities are required to further allocate resources, strengthen the research and development force of science and technology for China’s international education and the construction of education management team for overseas students to enhance the quality and equity of education.

Secondly, the combination of science and technology products and teaching was first applied to the education of foreign students and the education of Chinese-foreign cooperative schools. The wide use of scientific and technological products has accelerated the pace of China’s "going out", and the influence of "Introduce talents" has been enhanced [54]. It has enhanced international influence. In promoting cooperation between universities and enterprises, we can consider adopting the order cultivation mode to meet the needs of enterprises for high-end talents overseas, and actively carry out international enrollment of undergraduate education overseas, improve the quality of student teaching and training, and meet the needs of enterprises for high-end talents overseas.

Finally, it is clearly suggested that China’s international education should not only deepen and strengthen the innovation and application of emerging sciences and technologies such as artificial intelligence and 5G, fully tap the new educational needs in the epidemic period, and promote the iterative upgrading of teaching contents, forms and service tools of China’s international education, but also realize the mode of "Internet + education supervision" [55]. On the one hand, supervise the latest development of China’s international education science and technology, on the other hand, supervise the management of international education, so as not to fall into the strange circle of science and technology taking over everything. On July 19, 2021, the general office of the CPC Central Committee and the State Council issued a document, which requires to comprehensively reduce the burden of homework and after-school training for students in the stage of compulsory education, starts the strict governance of exam oriented education at the national level, and puts forward to strengthen quality education, requires to carry out rich and colorful popular science, sports, art, labor, reading, interest groups and community activities, and comprehensively check online education and training institutions, strictly control the quality, strengthen supervision and inspection, and establish a responsibility investigation mechanism. The signal released is to clearly reverse the serious adverse social impact of exam-oriented education from the root, build a high-quality education system, promote the equity of education and improve the quality of education. At the same time, it also shows the national supervision for higher education, including China’s international education.

The above policies and measures have injected a strong driving force into the improvement of quality, efficiency and scientific development of China’s international education, but it is also a kind of exploration. Compared with western countries, the road of innovation, development and application of young China’s international education science and technology is still very long, which needs continuous thinking, summary, exploration and practice.

### 5.3 Future development and research

Scientific and technological innovation of China’s international education is an important part of China’s higher education reform and scientific and technological development strategy. Therefore, the state and all sectors of society will be committed to its future deepening, improvement and scientific development. Based on the multi-level analysis and panoramic presentation of its current situation, this paper also studies and discusses its future development path. On this basis, China hopes to promote the common development of global education.

First of all, re-examine the development context of the times, strategic layout the innovation and application of China’s international education Science and technology. The long-term nature of COVID-19 provide the stage for the innovation and application of science and technology for China’s international education [56]. New changes in the global education industry and economic development pattern offer new opportunities for China’s international education [57]. The development law of China’s international education itself also promotes it to embark on the road of improving quality and efficiency. Therefore, China’s international education science and technology innovation needs to carry out systematic strategic layout and overall arrangement at the national level, or even the global level, to enhance the global mobility of students, rather than the current scattered or local propulsion. The forward-looking, overall and detailed operation strategy can coordinate the allocation of national resources, and can also strengthen the key areas or make up for deficiencies in weak areas.

Secondly, re-evaluate the educational needs and educational science and technology resources, and build a new ecology of international education. "In the future when the Internet, 5G technology and mobile terminals are highly developed, schools will become a learning community. In other words, it will be composed of a network learning center and an entity learning center to form a learning community. With the support of big data, blockchain and other technologies, establish a new credit bank. Individual international students come from different countries and regions, and their disciplines and majors are also different. Their national conditions, learning ideas, ways of thinking, culture, belief, professional background and other factors lead to different educational needs. In addition, the COVID-19 epidemic situation and the changing international situation, international education should re-evaluate the learning motivation and learning needs of international students under the new situation and the existing educational science and technology resources. On this basis, carry out science and technology innovation for international education, build a new ecology of international education with the advanced concept of educational science and technology, the teaching content of new core courses superimposed with personalized e-learning courses, online and offline mixed learning, man-machine combination or even man-machine cooperation, and the "intelligent + humanized" evaluation and assessment mechanism. At the same time, using advanced technologies such as big data and blockchain, establish a credit bank to break the connecting barrier between international students’ education in their own country and education in China, so as to maximize the seamless transfer of overseas students’ studies and the penetration and integration of knowledge system [58].

Finally, re-positioning the guarantee mechanism of scientific and technological innovation for international education, strengthening property rights protection and establishing a dynamic evaluation and supervision system. International education is a distinctive part of higher education, and its educational scientific and technological innovation and application are highly targeted. Therefore, be sure to strengthen the awareness of intellectual property protection, improve laws and regulations in this field, severely crack down on infringement and illegal acts, strengthen patent application, especially international invention patent application, and ensure the rights and interests of educational science and technology products. At the same time, establish a dynamic evaluation and supervision system to deal with the new trends, new needs and new problems in the process of rapid development and real-time change of international education, and realize the intelligent environment and new science education model of international education under the joint action of scientific evaluation and effective supervision, so as to improve the learning experience and satisfaction of international students, continuously strengthen the quality and international competitiveness of China’s international education.

## 6. Conclusion

In the context of the normalization of outbreak prevention and control, the application of higher education science and technology innovation plays a crucial role in the education of students studying in China. At the beginning of the 21st century, with the rapid advancement of science and technology, the education sector has gradually shifted to digitization and intelligence in order to enhance the quality and effectiveness of teaching and learning. However, the outbreak of the COVID-19 epidemic in 2020 brought unprecedented challenges to the world and accelerated changes in the education sector.

### 6.1 Implications of this study in theory and practice

Scientific and technological innovation in education is an important means of coping with public health emergencies. The outbreak of COVID-19 prompted the acceleration of scientific and technological innovation and application in the field of education, such as online classroom, virtual simulation teaching, etc., which have provided strong support to ensure the normal teaching and learning of education. This has inspired us to actively explore and utilize science and technology to improve the resilience and coping capacity of the education system in the face of similar challenges.

The application of science and technology in education needs to focus on its practicality and effectiveness. The material mentions that some education science and technology products have not been effectively developed for the learning and living environments and characteristics of Chinese students, resulting in insufficient application effectiveness. This tells us that in promoting the integration of science and technology in education, it is important to closely tie in with the actual needs and ensure that scientific and technological means can truly improve the quality of education.

### 6.2 Key lessons learned

Advance layout and strategic planning are crucial. The Chinese government’s "Internet+Education" strategy, which was laid out in advance during the COVID-19 outbreak, provided the basis for the large-scale implementation of online education. This shows that advance planning and strategic planning are key when facing future uncertainties and challenges.

The application of scientific and technological means needs to be diversified and personalized. The needs of students from different regions, disciplines and learning backgrounds for technological means are diversified. Therefore, in promoting the integration of science and technology with education, these differences need to be fully taken into account to provide personalized solutions.

Continuous innovation and improvement are the driving force behind the development of science and technology. The material mentions that although China has made certain achievements in education science and technology innovation, there are still some problems, such as not many high-end products, and the similarity of the attributes of some products. This tells us that scientific and technological innovation is a continuous process, which needs to be constantly pushed forward to overcome the existing problems and achieve better development.

### 6.3 Limitations of the research

The scope of the study is limited. This paper mainly focuses on the technological innovation and application of study abroad education in China during the COVID-19 epidemic, and it does not make an in-depth exploration of other countries and regions, so the scope of the study is somewhat limited.

Data testing is limited. The model was constructed through the construction process of clarifying the research objectives, defining the variables, analyzing the data using statistical methods, and supplementing with case arguments, but it still needs to be further tested and improved. Therefore, we will continue to optimize and improve our model in future research to better serve our research objectives.

Uncertainty of future development prediction. Although this paper has made certain predictions and analyzed the future development of study abroad education, the prediction results have a certain degree of uncertainty because scientific and technological innovation in the field of education is a complex process, which is affected by a variety of factors.

In general, through the practical application of education technology innovation in the education of studying abroad in China, it can be seen that although there are still some shortcomings in the application of education technology innovation in China, with the continuous progress and improvement of technology, we believe that China will be able to achieve more significant results in the application of education technology innovation in the future.

Finally, propose partially actionable measures and recommendations based on findings: On the one hand, establish a risk assessment and response mechanism for study abroad education. Establish a risk assessment system for study abroad education, and make a comprehensive assessment of the market, policy, economy and other aspects of study abroad education. Formulate a risk response plan and disposal mechanism for study abroad education, strengthen campus safety management, ensure campus stability and the safety of teachers and students, and ensure rapid response and problem solving in case of risks. Strengthen cooperation and exchanges with international organizations and educational institutions to jointly address global educational challenges and risks. On the other hand, promote the digitization and informatization of education. Strengthen the construction of online education platforms and resources, and improve the quality and effectiveness of online teaching. Promote the construction of digital campuses to realize information sharing and convenient services. Strengthen digital cooperation with international educational institutions and jointly promote the construction of education digitization and informatization. It can effectively promote the sustainable and healthy development of education for studying in China.
